# Sources of Individual Differences in Children’s Matrix Problem-Solving Abilities: Evidence from Eye Movements

**DOI:** 10.11114/jets.v14i1.8056

**Published:** 2025-12-27

**Authors:** Brenda A.M. Hannon

**Affiliations:** Department of Psychology, Texas A&M University – Kingsville, USA

**Keywords:** children, problem solving, matrix reasoning, individual differences

## Abstract

This study employs a novel combination of eye-tracking technology and matrix completion problems to investigate some of the sources of individual differences in problem-solving skills among 67 children aged 7–8 years. Our study provides physiological evidence that children who are better problem solvers examine the matrix areas of matrix completion problems longer than response-choice areas; a finding that suggests they are most likely adopting a constructive matching approach for solving problems. In contrast, poor problem solvers examine the response-choice areas longer than better problem solvers. They also examine the matrices for a considerable amount of time after viewing the response choices. These findings suggest that poor problem solvers are more likely to adopt a response-elimination approach for solving problems than better problem solvers. Finally, our study shows that children who are better problem solvers systematically study the rows and columns in the matrices more frequently than poor problem solvers. This latter finding suggests that better problem solvers intentionally try to extract the underlying structural features of the matrix completion problems.

## 1. Introduction

**Figure 1.**
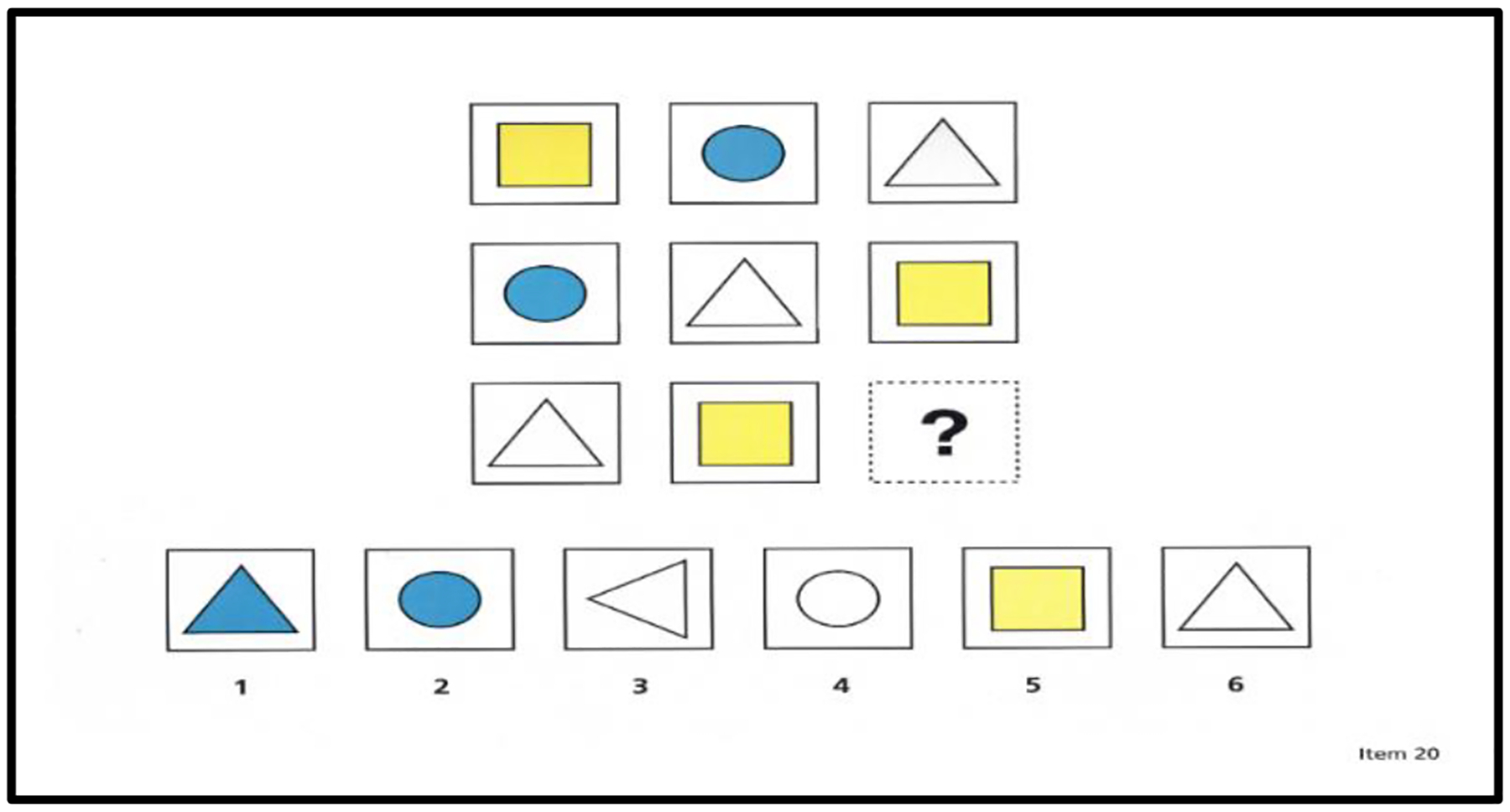
Sample matrix completion problem

Matrix completion tasks are among the most common measures of reasoning, as evidenced by their inclusion in cognitive assessment batteries and their application in fluid intelligence research (Perret & Dauvier, 2018); see Figure 1 for an example. For these and other reasons, researchers have sought to identify factors contributing to individual differences in matrix completion tasks. Most researchers have used conventional paper-and-pencil versions of these tasks. However, a few researchers (e.g., Vigneau et al., 2006) have used eye-tracking technology to record the eye movements of individuals as they complete the problems. The advantage of eye tracking is that it provides direct, physiological evidence of factors contributing to skill differences in matrix problem solving, such as differences in strategy choices (e.g., Gonthier et al., 2024; Laurence & Macedo, 2023). Eye tracking research has also led to the proposal of a multifaceted model of intelligence (e.g., Vigneau et al., 2006) in which individual differences in intelligence are explained in terms of factors, such as processing speed (e.g., Kail, 2007), working memory (e.g., Gonthier & Roulin, 2020), relational reasoning (e.g., Dauvier et al., 2014; Resing et al., 2017), and strategic influences (e.g., Vigneau et al., 2006).

The present study employs a novel combination of eye-tracking technology and matrix completion problems to identify some of the sources of individual differences in the problem-solving skills of young children. Of particular interest is the amount of time children spend studying matrix areas of the completion problems (i.e., matrix areas of interest or matrix AOIs), the amount of time children spend studying response-choice areas of the completion problems (i.e., response-choice areas of interest or response-choice AOIs), and children’s attempts to uncover the patterns or *structural features* of the matrices. Do children who are better problem solvers mirror their adult counterparts by studying matrix AOIs longer than response-choice AOIs? And do children who are better problem solvers purposely try to uncover the underlying structural features of a matrix more frequently than children who are poor problem solvers? Below, we discuss relevant problem-solving literature, followed by the present study.

Matrix completion problems include two main areas of interest (i.e., AOIs): matrix AOIs and response-choice AOIs; see Figure 1 for an example. Each matrix AOI includes multiple cells depicting different shapes, except for one empty cell with a question mark in the bottom right corner of the matrix. Each response-choice AOI includes five alternative response choices. An individual’s task is to study a matrix and select the response choice that best fits the empty cell with the question mark. Ideally, to complete a matrix problem, an individual must uncover the unique logical rules underlying the matrix; one set of logical rules underlie the rows and one set of logical rules underlie the columns (Gonthier et al., 2024). These underlying logical rules are induced by comparing differences among the cells in the rows and among the cells in the columns (Dauvier et al., 2014). This comparison process is often described as relational reasoning (Resing et al., 2017).

Over the past few decades, some researchers have sought to uncover the strategies that individuals execute when solving matrix completion problems (Laurence & Macedo, 2023). The strategies most generally accepted within the research community are Snow’s (1978, 1980) and Bethell-Fox et al.’s (1984) *constructive matching* and *response elimination strategies* (Laurence & Macedo, 2023). Of these two strategies, constructive matching is considered more successful (e.g., Gonthier et al., 2024). The constructive matching strategy involves constructing or forming an idealized answer using the cells in a matrix AOI before viewing the response alternatives (Bethell-Fox et al., 1984; Perret & Dauvier, 2018). After forming an idealized answer, an individual searches the response alternatives for an alternative that most resembles their idealized answer (Laurence & Macedo, 2023). In contrast, the response elimination strategy involves an individual spending the bulk of their time comparing response alternatives in the response-choice AOIs to cells in a matrix (Bethell-Fox et al., 1984).

The trade-off between these two strategies has been observed in the gaze patterns of adults as they complete Raven’s matrices, a common matrix completion task. In adult research, longer latencies on the matrix AOIs than the response-choice AOIs and longer latencies on the matrix AOIs before toggling to response-choice AOIs are both considered as evidence that the problem solver adopted a constructive matching strategy (Gonthier et al., 2024; Vigneau et al., 2024). These gaze patterns are also hallmarks of better problem-solving skills (Matlin, 1994; Sternberg, 1977). On the other hand, toggling more frequently between matrix and response-choice AOIs, shorter latencies on the matrix AOIs, and shorter latencies on the matrix AOIs before toggling to response-choice AOIs are considered as evidence that the problem solver adopted a response elimination strategy (Gonthier et al., 2024; Laurence & Macedo, 2023; Vigneau et al., 2024). They are also evidence of poorer problem-solving skills (Matlin, 1994; Sternberg, 1977). Vigneau et al. (2006), for example, observed that better adult problem solvers studied matrix AOIs longer than response-choice AOIs, whereas poor problem solvers studied response-choice AOIs longer than matrix AOIs. Presumably, better problem solvers spent more time constructing an idealized answer for a matrix before considering the response alternatives. On the other hand, better problem solvers were less likely than poor problem solvers to toggle between the matrix and response-choice AOIs, suggesting that poor problem solvers adopted a response-elimination strategy. Finally, in comparison to poor problem solvers, better problem solvers spent more time during their initial inspections of the matrix AOIs before toggling to the response-choice AOIs (Vigneau et al., 2006); another finding that suggests better problem solvers adopted a constructive matching strategy. This latter finding is also physiological evidence for a classic finding in problem-solving research: Better problem solvers spend more time than poor problem solvers during the encoding phases of reasoning problems (e.g., Lesgold, 1988; Sternberg, 1977; Voss & Post, 1988).

Research targeting children also provides insights into sources of individual differences in problem-solving skills, especially regarding constructive matching and response elimination strategies (e.g., Chen et al., 2016; Gonthier et al., 2024). For example, Chen et al.’s eye-tracking study (2016) presented children with 3 × 3 matrix completion problems that included two dimensions (i.e., shape, color, size, orientation); one dimension for the rows and another for the columns. Of particular interest were measures that assessed the number of problems in which children viewed: (i) all three cells of either a row or a column (i.e., *encoding*), and (ii) all three cells in a row and all three cells in a column (i.e., *integration*) because these two measures presumably assessed whether children used relational reasoning to induce the logical rules underlying the rows and/or columns (Chen et al., 2016). Higher encoding and integration scores indicated that children adopted a constructive matching strategy, especially for the integration measure, which considered the logical rules underlying both the rows and the columns in a matrix. In addition, Chen et al. assessed the number of toggles between the matrix and response-choice AOIs; a higher number of toggles indicated that children employed a response-elimination strategy. Chen et al. observed that both encoding and integration were positively related to overall performance on the matrix completion task, *r* = .60 and *r* = .37 respectively. These findings suggest that inducing the logical rule(s) underlying the rows and columns leads to better performance. They also support the idea that a constructive matching strategy leads to better performance. In addition, Chen et al. observed that for 7- and 8-year-olds, there was an equivalent number of toggles between matrix and response-choice AOIs for better and poor problem solvers. This null finding provides no evidence for skill differences in toggling between the matrix and response-choice AOIs. It also does not rule out the possibility that children use a response elimination strategy, especially given that Chen et al. observed a positive relationship between overall performance and toggling between the matrix and response-choice AOIs, *r* = .41.

Chen et al.’s (2016) finding concerning children viewing the logical rules of matrices (i.e., encoding and integration) complements a seminal finding in the adult literature. This finding is that there are large skill differences in the degree to which adult problem solvers use underlying logical rules or *structural features* to solve a problem (Novick, 1988). In particular, research suggests that whereas better adult problem solvers rely more heavily on a matrix’s underlying logical rules or structural features, poor adult problem solvers rely more heavily on a matrix’s surface features (e.g., Matlin, 1994; Novick, 1988), such as color. In the context of matrix completion problems, this means that better problem solvers will rely more heavily on changes in the patterns from row to row and column to column (i.e., changes in the underlying structural features). Research also suggests that better adult problem solvers are more likely to transfer the structural features to similar problems (e.g., Novick, 1988) and form representations of problems using abstract ideas (e.g., Larkin, 1983; 1985). In contrast, poor problem solvers frequently fail to transfer structural features to similar problems (e.g., Novick, 1988) and form representations using objects from the real world (e.g., Larkin, 1983; 1985). This skill difference is so striking that some researchers have postulated that a core difference between better and poor problem solvers is the degree to which they use structural features (Carpenter et al., 1990).

### 1.1 Current Study

Building on the work of Vigneau et al. (2006), Chen et al. (2016), and Novick (1988), the present study aimed to provide direct evidence of the factors contributing to children’s skill differences in matrix completion problems. For this reason, we used eye-tracking technology to obtain real-time “online” information about children as they solved matrix completion problems. As in Vigneau et al., we collected latencies for matrix AOIs, response-choice AOIs, and the matrix AOIs before toggling to the response AOIs. As in Chen et al. (2016), we computed the number of problems in which a child viewed all three cells in at least one row or column (i.e., encoding), the number of problems in which a child encoded at least all three cells in one row and at least all three cells in one column (i.e., integration), and the average number of toggles a child made between the matrix and response-choice AOIs. Blending the measures of these two studies enables us to utilize the latency measures from Vigneau et al.’s adult study to support the non-latency measures from Chen et al.’s children’s study, and vice versa. Finally, based on Novick’s work, we developed a novel measure that assesses the extent to which children attempt to uncover the underlying logical rules or structural features of matrix completion problems. However, unlike Chen et al.’s measures of encoding and integration, which assessed whether a child did or did not encode or integrate a problem, this new measure assesses the frequency or extent to which a child searches for the underlying rules for the rows and/or columns of a matrix.

#### 1.1.1 Hypotheses

Including all of these measures (i.e., Vigneau et al.’s (2006), Chen et al.’s (2016), and ours) allowed us to uncover some of the sources of individual differences in children’s problem-solving skills. It also allowed us to determine whether children mirror adult problem solvers. For example, by separating the time children study the matrix AOIs versus the response-choice AOIs, we can determine whether children mirror adult problem solvers’ use of information from the matrix and the response-choice AOIs. Do children who are better problem solvers study matrix AOIs longer than poor problem solvers? Do better problem solvers study matrix AOIs longer than response-choice AOIs? Do poor problem solvers study the response-choice AOIs longer than the matrix AOIs?

##### Hypothesis 1 (H1):

If children mirror adults, then, based on the findings of Vigneau et al., children who are better problem solvers will study the matrix AOIs longer than the response-choice AOIs. In contrast, children who are poor problem solvers will study the response-choice AOIs longer than the matrix AOIs.

**Figure 2.**
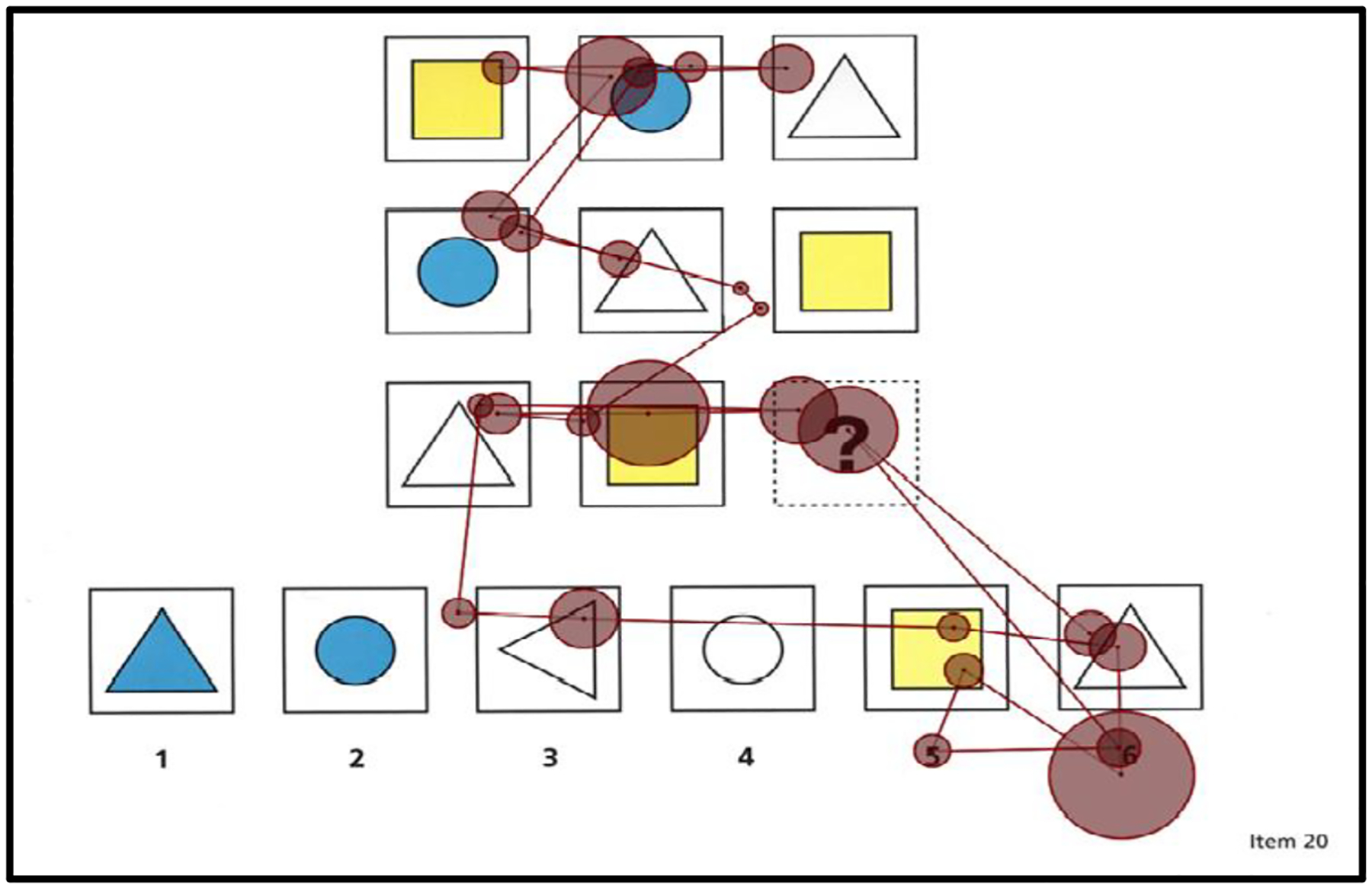
An example scan path of a child studying a matrix completion problem

To assess the extent to which children use the underlying logical rules or structural features of matrix completion problems, we used Chen et al.’s (2016) encoding and integration measures as well as our new measure of rows and columns. For our rows and columns measure, we tallied the number of rows and columns that children systematically studied in a matrix; see Figure 2 for an example. Our rationale for this measure was based on three essential principles in problem-solving: (i) identifying patterns in rows and columns provides important information about the underlying structural features of a matrix (e.g., Dauvier et al., 2014; Novick, 1988; Resing et al., 2017), (ii) knowing the underlying structural features of a matrix is important for solving a matrix problem (e.g., Dauvier et al., 2014; Novick, 1988; Resing et al., 2017), and (iii) use of the rows and columns in a matrix to solve a problem will increase as a function of problem-solving skill given that better problem solvers have a greater appreciation of the importance of a matrix’s underlying structure (e.g., Novick, 1988).

##### Hypothesis 2 (H2):

Based on the findings of adult research (e.g., Novick, 1988), we hypothesized that children who are better problem solvers will study rows and columns in the matrices more frequently than children who are poor problem solvers.

##### Hypothesis 3 (H3):

Based on the findings of problem-solving research targeting children (e.g., Chen et al., 2016), we hypothesized that children who are better problem solvers will encode and integrate more problems than poor problem solvers.

## 2. Methods

### 2.1 Participants

We recruited 67 children from a local school. The 34 girls and 33 boys ranged in age from 6.67 to 8.03 years, *mean age* = 7.31, *S.D*. = 0.31. Fifty-four of the children were of Hispanic descent, while the remaining 13 were of European American, Asian, or African American descent. All children were dominant English speakers, which we verified by asking the children’s parents and the teachers. All children received a toy package as remuneration for their participation in our study.

### 2.2 Matrix Completion Task

#### 2.2.1 Materials

The matrix completion task was a self-paced, computerized version of the matrix subtest of the Cognitive Assessment System (CAS2: Naglieri & Das, 1997). The CAS2 matrix subtest includes 1 practice problem and 44 critical problems, incrementing in difficulty with each successive problem. See Figure 1 for an example.

The CAS2 and its subtests are suitable for children aged 5 to 18. The matrix subtest of the CAS2, which is the subtest of interest in our study, was constructed using problems that conform to those included in the Matrix Analogies Test (Naglieri, 1985) and the Naglieri Nonverbal Abilities Test (Naglieri, 1996). According to the Interpretive and Technical Manual for the CAS2, to arrive at a correct solution for a problem, children must recognize the relationships between the component parts of a problem (Naglieri & Das, 1997, p. 78). In addition, problems progress from relatively simple to complex, such that later problems become more multidimensional and require the synthesis of complex relationships (Naglieri & Das, 1997, p.78).

The matrix completion subtest and other CAS2 subtests have a .80 correlation with the IQ measure from the Wechsler Intelligence Scale for Children, Third Edition (i.e., WISC-III, Parvaneh et al., 2010), and a .77 correlation with the general fluid intelligence measure from the Woodcock-Johnson III (i.e., WJ-III, Keith et al., 2001). The Cronbach alpha for 8-year-olds is .88.

In our computerized version of this matrix completion task, we made every effort to ensure that the digitized versions of the practice and critical problems were equivalent to their pencil-and-paper counterparts. For example, the sizes of the computerized problems corresponded to those of their pencil-and-paper counterparts, and both the matrices and response choices were presented simultaneously on the computer screen.

#### 2.2.2 Eye-movement Tracking

The computerized practice and critical problems were integrated into a SMI RED-m eye-tracking system, which sampled and recorded the fixations and movements of a child’s pupils as they completed each matrix completion problem. The RED-m eye-tracking system consists of a high-powered binocular 120 Hz camera unit. Although the camera unit tracks the movements of both eyes, the recordings and subsequent measures reported in the present study are based on data from the best-calibrated eye (i.e., monocular). The RED-m eye-tracking system uses pupil and marker positions to compute gaze position with an average accuracy of 0.4° or better. Finally, the RED-m eye-tracking system provides three types of recordings simultaneously, namely, a video recording of the child from the waist up, an auditory recording of what a child says, and a recording of a child’s eye movements. These multiple recordings help verify a child’s answers and identify a child’s pattern of eye movements while they complete the matrix problems.

#### 2.2.3 Procedure

The computer monitor, the child’s chair, and the eye-tracking camera unit were adjusted until they conformed to the RED-m eye-tracking system recommendation that a participant sit with their face 60–80 cm away from the computer monitor of the eye-tracking system. At this point, the experimenter performed calibration and validation procedures following the standards set out by the SMI RED-m eye-tracking software. The experimenter repeated these calibration and validation procedures until satisfactory levels were achieved.

Next, the experimenter read the instructions for the CAS2 matrix completion task to the child as they studied the matrix and response choices for the practice problem on the computer monitor. After studying the practice problem, the child reported their answer aloud. At this point, the experimenter pressed the spacebar on a keyboard, which stopped the eye movement recording of the practice problem and initiated the presentation of a blue screen with a white asterisk in the middle of the computer screen. This blue screen remained on the computer monitor while the experimenter recorded the child’s answer. After recording the answer, the experimenter verified that the child was ready to continue, instructed the child to fixate on the white asterisk on the computer screen, and then pressed the spacebar, which initiated both the presentation of the next matrix completion problem and started the recording of the eye movements. This matrix problem-then-answer procedure was repeated until the child answered four successive problems incorrectly. At this point, the experimenter discontinued the matrix completion task. The total time to complete this task depended on the number of problems a child solved. The more problems a child completed, the longer the task.

For the eye movement data, we analyzed only the first 22 problems of the matrix completion task because while all children in the present study completed the first 22 problems, not all children completed 23 or more problems. Using 22 problems as our cutoff, we based our analysis on the same 22 problems for all children, thereby avoiding confounding our results by using different problems for different children. The classification of problem-solving skills was also based on the first 22 problems.

#### 2.2.4 Matrix Completion Task Eye-movement Measures

**Figure 3.**
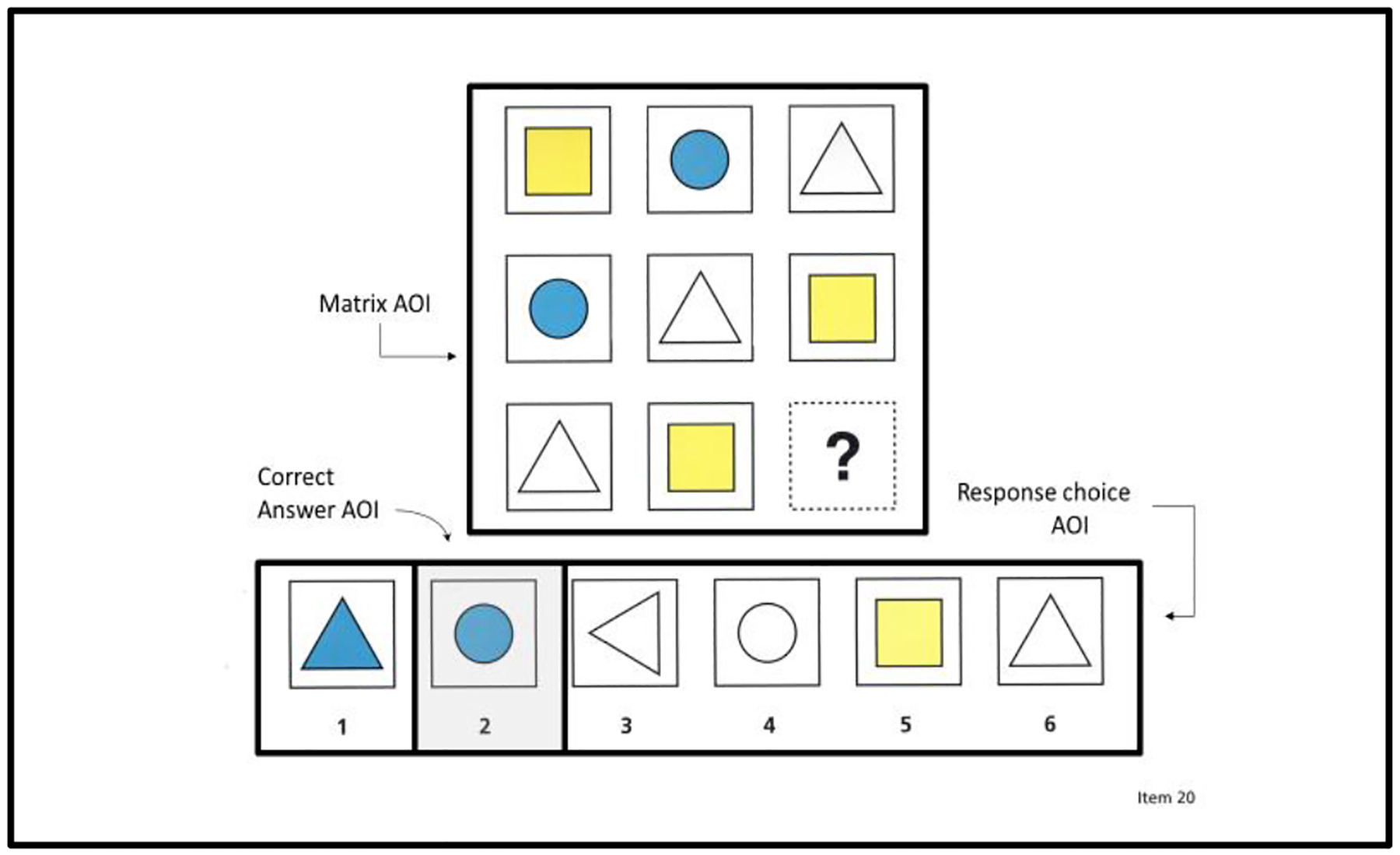
Areas of Interest: Matrix AOI, response-choice AOI, and correct answer AOI

The SMI RED-m software computed the eye-movement measures relating to the latency of an AOI (i.e., mean matrix latency, mean response-choice latency, mean correct answer latency) and the initial latency of a matrix AOI before switching to another area of the matrix completion problem (i.e., mean latency-before-first-toggle). Measures assessing latencies for AOIs (i.e., mean matrix latency, mean response-choice latency, mean correct answer latency) were limited to eye movements within the perimeter of that respective AOI. Figure 3 depicts the perimeters for the matrix, the response choice, and the correct answer AOIs. It should be noted that the correct answer AOI was within the response-choice AOI. So, the latency for the response-choice AOI included both the latencies for the incorrect answer choices and the correct answer choice.

We also created a measure called the mean number of rows and columns, the average number of rows and columns a child studied in the matrix AOIs. Below, we describe, in detail, our measures assessing AOI latency, latency-to-first-toggle, toggling, encoding, integration, and the number of rows and columns.

##### 2.2.4.1 Mean AOI Latencies and Mean Latency

A matrix latency for any given problem was the total time a child’s gaze was within the perimeter of a matrix AOI. *Mean matrix latency* was the average latency for the 22 matrix AOIs. A response-choice latency for any given problem was the total time a child’s gaze was within the perimeter of a response-choice AOI*. Mean response-choice latency* was the average latency for the 22 response-choice AOIs. A correct answer latency for any given problem was the total time a child’s gaze was within the perimeter of a correct answer AOI. *Mean correct answer latency* was the average latency for the 22 correct answer AOIs. Finally, a total latency for any given problem was the total time a child’s gaze was within the perimeters of both the matrix and response-choice AOIs. The *mean total latency* was the average latency for the 22 total latencies.

A matrix latency-before-first-toggle for any given problem was the total time a child’s gaze was focused within the perimeter of a matrix AOI before switching or toggling to another AOI of that same matrix completion problem. *Mean matrix latency-before-first-toggle* was the average time a child’s gaze was within the perimeters of the 22 matrix AOIs before toggling to other areas of the matrix problems. A matrix latency-after-first-toggle for any given problem was the total time a child’s gaze was focused within the perimeter of a matrix AOI after switching or toggling to another AOI of that same matrix completion problem. *Mean matrix latency-after-first-toggle* was the average time a child’s gaze was within the perimeters of the 22 matrix AOIs after toggling to another area of the matrix problems. Finally, an incorrect response-choice latency for any given problem was the total time a child’s gaze was on response choices that were incorrect. We calculated the incorrect response-choice latency by subtracting the latency for the correct answer AOI from the latency for the response-choice AOI, which was a composite measure of all response choices, correct and incorrect (i.e., incorrect response-choice latency = response-choice AOI latency – correct answer AOI). *Mean incorrect response-choice latency* was the average of a child’s 22 incorrect response-choice latencies.

We also generated six standardized measures by dividing each AOI latency by total latency. These standardized measures provided information about how individuals proportionally spend their time among the AOIs. The proportion of time on the matrix for any given problem was the total time a child’s gaze was focused within the perimeter of a matrix AOI divided by that matrix’s total latency (i.e., matrix AOI / total latency). *Mean proportional time on matrix* was the average proportion of time a child’s gaze was within the perimeters of the 22 matrices. The proportion of time on the matrix before the first toggle for any given problem was the total time a child’s gaze was focused within the perimeter of a matrix AOI before toggling to another AOI, divided by that matrix’s total latency. *Mean proportional time on matrix before first toggle* was the average proportion of time a child’s gaze was within the perimeters of the 22 matrices before toggling to other AOIs. The proportion of time on the matrix after the first toggle for any given problem was the total time a child’s gaze was focused within the perimeter of a matrix AOI after toggling to another AOI, divided by that matrix’s total latency. *Mean proportional time on matrix after first toggle* was the average proportion of time a child’s gaze was within the perimeters of the 22 matrices after toggling to other AOIs. The proportion of time on response choices for any given problem was the total time a child’s gaze was focused within the perimeter of a response-choice AOI divided by that matrix’s total latency. *Mean proportion time on response choices* was the average time a child’s gaze was within the perimeters of the 22 response-choice AOIs. The proportion of time on correct response choices for any given problem was the total time a child’s gaze was focused within the perimeter of a correct answer AOI divided by that matrix’s total latency. *The mean proportion of time on correct answer choices* was the average proportion of time a child’s gaze was within the perimeters of the 22 correct answer AOIs. The proportion of time on incorrect response choices was calculated by subtracting the latency for the correct answer AOI from the latency for the response-choice AOI divided by that matrix’s total latency (i.e., (response-choice AOI – correct-answer AOI)/total latency). *Mean proportion incorrect response-choice is* the average of a child’s 22 incorrect proportion response-choices.

##### 2.2.4.2 Mean Number of Toggles and Mean Rate of Toggling

The number of toggles for any given problem was the total number of times a child gazed back and forth between the matrix and response-choices AOIs (Vigneau et al., 2006). The *mean number of toggles* was the average number of toggles a child made on the 22 problems. We also standardized the measure for the number of toggles by using a problem’s latency to create a rate of toggling measure (i.e., number of toggles for a problem/latency for that problem). *The mean rate of toggling was the average* rate of toggling for the 22 problems.

##### 2.2.4.3 Encoding and Integration^Note 1^

We used 17 of the first 22 problems to compute: (i) the number of problems in which a child encoded at least one row or column, and (ii) the number of problems in which a child integrated at least one row and column. For the measure of *encoding*, as in Chen et al. (2016), a row was deemed encoded if the child’s gaze scanned completely across at least one row from left to right or right to left, or if a child’s gaze completely scanned up and down or down and up at least one column. For the measure of *integration*, as in Chen et al., a problem was considered “integrated” if a child’s gaze scanned completely across a row from left to right or right to left and scanned completely up and down or down and up a column.

##### 2.2.4.4 Mean Number of Rows and Columns

We used 17 of the first 22 problems to compute the mean number of rows and columns. *The mean number of rows and columns* was the average number of rows and columns a child studied in a matrix AOI. The mean number of rows and columns was determined by three independent raters who were naïve about the problem-solving skill levels of the children. The first two raters independently computed each child’s mean of rows and columns. The third rater reconciled any differences between the means computed by the first two raters. Figure 4(i) shows examples of what we operationalized as a row, and Figure 4(ii) shows examples of what we operationalized as a column. As Figure 4(i) shows, when a child completed either a full left-to-right/right-to-left scan of a matrix or a part left-to-right/right-to-left scan of a matrix (part = scan of two cells), it was counted as a row. Similarly, as Figure 4(ii) shows, when a child completed a full up-down/down-up scan of a matrix or a part up-down/down-up scan of a matrix (part = scan of two cells), it was counted as a column. Our rationale for giving credit for part scans was that part scans still show that a child is trying to uncover the underlying pattern of a row or column. Figure 2 shows a child’s scan path for a matrix completion problem. This figure shows that this child scanned full and partial rows and columns multiple times.

#### 2.2.5 Matrix Completion Task Accuracy

We used the first 22 problems to compute accuracy. *Accuracy* was measured by the total number of problems answered correctly. Accuracy ranged from 14 to 22.

##### 2.2.5.1 Problem-solving Skill Level

Problem-solving skills were based on performance on the 22 problems that all children completed. Poor problem solvers had scores < 19, and better problem solvers had scores > 18. We chose the adjective ‘better’ rather than ‘skilled’ or ‘expert’ to describe children with the highest problem-solving skills because we interpret “skilled” and ‘expert’ as adjectives that are associated with problem-solving experiences that children lack (Matlin, 1994). Given the age of our children, it is safe to say that our problem solvers did not hone their skills through years of practice.

## 3. Results

Table 1 reports the correlations and descriptive statistics for accuracy and the eye-movement measures. To reiterate, accuracy is the number of problems children answered correctly. The mean latency was the average time it took a child to view a matrix problem. Mean matrix latency, mean response-choice latency, and mean correct latency were the average times a child’s gaze was within the perimeters of the matrix AOIs, the response-choice AOIs, and the correct answer AOIs, respectively. Mean matrix latency-before-first-toggle was the average time a child’s gaze was within the perimeter of a matrix AOI before toggling to another area of that same matrix completion problem. Mean matrix latency-after-first-toggle was the average time that a child’s gaze was in the perimeter of a matrix AOI after toggling to another AOI.

We also calculated incorrect response-choice latency, which was the amount of time a child spent viewing incorrect answers, and proportion measures for our six matrix and response-choice latencies. The mean number of toggles was the average number of times a child gazed back and forth between the matrix and response-choices AOIs. Encoding was the number of problems that a child scanned a complete row or column, whereas integration was the number of problems that a child scanned a complete row and column. Finally, the mean number of rows and columns was the average number of rows and columns a child studied in a matrix AOI.

Below, we report the results of our item analyses, which showed that later problems became more difficult to complete, especially the last 11 problems. For the subjects’ data, we report the correlations among the measures, which showed that the mean matrix latency, mean matrix latency before the first toggle, integration, and the mean number of rows and columns correlated positively with accuracy. The number of rows and columns was the strongest predictor of these four measures. We also conducted a series of t-tests, which revealed that for better problem solvers, latencies on matrix AOIs were significantly longer than those for response choices. In contrast, the latencies for poor problem solvers were the same for matrices and response choices. Moreover, their latencies for matrix AOIs after toggling to other AOIs were greater than those for matrix AOIs before toggling to other AOIs.

### 3.1 Item Analysis

Accuracy was converted to percentages to streamline the assessment of problem difficulty. Higher percentages indicate easier problems, and lower percentages indicate more difficult problems. The analysis revealed that as the problem number increased, the percentage of students who answered the problems correctly decreased, from 97% to 30%, *r* = −.80. This finding suggests that difficulty increased with successive problems. However, further investigation revealed that the accuracy for the first 11 problems was very high, *with a range of* 94% to 100%, which suggests a ceiling effect. Subsequent analysis revealed that the −.80 correlation was because of large decreases in accuracy for the latter 11 problems, *r* = −.94, and not for large declines in accuracy for the first 11 problems, *r* = −.32. In retrospect, this latter finding is not surprising given that the CAS2 matrix subtest was designed for children as young as 5 (Naglieri & Das, 1997). And although the earlier problems may differentiate the performances of children < 7 years old, clearly they do not differentiate the performances of children in the present study, who are approximately 7.31 years old.

Mean problem latency also strongly correlated with problems, with children spending more time on later, more difficult problems, *r* = .77. Additionally, mean matrix latency and mean matrix latency before first toggle correlated strongly with problems, *r* = .74 and *r* =.70, with children viewing matrix AOIs longer for later, more difficult problems. Similarly, the proportion of time on the matrix correlated strongly with problems, *r* = .58, which suggests that children viewed matrix AOIs proportionally longer on later, more difficult problems. However, even though children viewed the matrix AOIs proportionally longer on more difficult problems, the proportion of time on the matrix before the first toggle showed little correlation with problems, *r* = .21, while the proportion of time on the matrix after the first toggle correlated well, *r* = .64. Supplementary analysis, which calculated the proportion of time on the matrix before the first toggle and the proportion of time on the matrix after first toggle using matrix latency as the denominator instead of total latency, revealed that as the problem difficulty increased, children proportionally decreased their time on the matrices before viewing the response choices, *r* = −.45 and proportionally increased their time on the matrices after viewing the response choices, *r* = .43. Taken as a whole, the three proportion measures for the matrix (i.e., proportion time on matrix, proportion time on matrix before first toggle, and proportion time on matrix after first toggle), suggest that although children increased the amount of time they spent on the matrices as the problems become more difficult, this additional time was spent after looking at the response choices. What is unclear is whether this finding applies to all children or just poor problem solvers.

In addition, and as expected, for mean response-choice latency, children spent more time on later, more difficult problems, *r* = .62. Interestingly, there was no change in mean correct answer latency across the 22 problems, *r* = .01, which suggests children spent the same amount of time viewing the correct answer for earlier, easy problems and later, difficult problems. This null finding also indicates that the .62 correlation we observed for mean response-choice latency is likely a consequence of latencies attributed to viewing incorrect response-choices and not latencies for combined correct and incorrect response-choices (i.e., response choice latency). To verify this supposition, we calculated a measure of mean incorrect response-choice latency and correlated latencies on this measure with the problems. The results revealed a .70 correlation, which suggests children spend more time viewing incorrect response choices for later, more difficult problems.

When the three standardized measures for response latency were considered, the analysis revealed that children spent proportionally less time on the response choices as the problem became more difficult, *r* = −.58. This decrease was a consequence of children spending proportionally less time viewing correct answer choices, *r* = −.76, and slightly more time viewing incorrect answer choices, *r* = .28. These latter correlations are not surprising given that children did not increase their viewing time for correct answer choices as the problem difficulty increased, *r* = .01, but increased their viewing time for incorrect answer choices, *r* = .70.

For toggling, children toggled more on later, more difficult problems than on earlier, easier problems, r = .62. This finding suggests that children tended to adopt a response elimination strategy when the problems became more difficult. When the number of toggles was standardized using problem latency to create a measure of mean rate of toggling, children still spent more time on more difficult problems, *r* = .70. This finding confirms our observations and interpretation for the mean number of toggles.

Finally, children tended to encode and integrate later, more difficult problems than earlier, easy problems, *r* = .60 and *r* = .53 respectively, two findings that suggest children tended to adopt a constructive matching strategy for later, more difficult problems. Finally, the mean number of rows and columns that children viewed increased with problem difficulty, *r* = .77, a finding that suggests that children scanned rows and columns more frequently on later, more difficult problems.

### 3.2 Descriptive Statistics

Table 1 presents the means, standard deviations, skewness, kurtosis, and Cronbach’s alphas for the measures in our study. As this table shows, all the measures had wide ranges and normal distributions (i.e., all values for skew and kurtosis were well below 3.00 and above −3.00. With respect to Cronbach’s alphas, statisticians have debated what constitutes an acceptable size for Cronbach’s alpha (Vaske et al., 2017, p. 165). By convention, an alpha of .65 to .80 is often considered “adequate” for a scale used in human research (Vaske et al., 2017, p.165). However, lower alphas may be acceptable in exploratory research using newer measures. Using all of these guidelines, many of the measures in the present study have acceptable alphas. Nevertheless, the mean matrix latency after first toggle, the mean proportion time on matrix after first toggle, the mean proportion time on incorrect response choices, the mean rate of toggling, encoding, and integration measures all have poor Cronbach alphas. Because of their poor alphas, the results of these measures will be downplayed, and supplementary analysis will be used where possible.

### 3.3 Correlational Analysis

Table 1 reports the correlations among the measures. As noted earlier, the measures were a combination of the latency eye-tracking measures used in Vigneau et al.’s adult study (2006) and the non-latency measures used in Chen et al.’s study (2016). We also included a measure of the average number of rows and columns that children examined in a matrix.

Do better problem solvers study matrix AOIs longer than poor problem solvers? The answer to this question is *yes*. As Table 1 shows, accuracy was positively related to mean matrix latency; a finding that suggests better problem solvers examined the matrices longer than poor problem solvers, *r* = .35. Further investigation revealed that the source of this .35 correlation was attributed to the time problem solvers studied the matrices before the first toggle, *r* = .45 and not the time they spent studying the matrices after the first toggle, *r* = .05. Taken as a whole, these findings are consistent with adult research (Vigneau et al., 2006), and provide physiological evidence for a classic finding in problem-solving research, namely: Better problem solvers spend more time than poor problem solvers during the encoding phrases of reasoning tasks (e.g., Lesgold, 1988; Sternberg, 1977; Voss & Post, 1988).

Do poor problem solvers study the response-choice AOIs longer than better problem solvers? The answer to this question is *maybe*. As Table 1 shows, there were no relationships between accuracy and mean response-choice latency, *r* =.05, and between accuracy and mean correct answer latency, *r* = −.08. These findings suggest that mean response-choice latency and mean correct answer latency are equivalent for better and poor problem solvers. On the other hand, accuracy and incorrect response-choice latency were marginally correlated, *r* = −.21, *p* < .09, a finding that suggests poor problem solvers tended to spend more time on incorrect response choices than better problem solvers.

Do poor problem solvers toggle between the matrix and the response-choice AOIs more so than better problem solvers? The answer to this question is *no*. As Table 1 shows, there was no relationship between children’s accuracy on the problems and toggling between the matrix and response-choice AOIs, *r* = .06. Nor was there any relationship between accuracy and rate of toggling, a standardized measure of toggling, *r* = .18. These findings replicate those of Chen et al. (2016), who showed that toggling was not predictive of accuracy on matrix problems for 7- and 8-year-olds. More importantly, these findings, in conjunction with the item analyses, which showed that toggling increased as problem difficulty increased, suggest that both better and poor problem solvers increase their toggling as problems get more difficult.

Do the number of problems in which a child scans either a row or column (i.e., encoding), the number of problems in which a child scans both a row and a column (i.e., integration), and/or the number of rows and columns a child examines in a matrix relate to a child’s accuracy on the matrix completion task? The answer to this question is *no* for encoding, *yes* for integration, and *yes* for the number of rows and columns. As Table 1 shows, the number of problems in which encoding occurred was not related to accuracy on the matrix completion task, *r* = .18. This finding not only fails to support Hypothesis 2 but it also fails to replicate those of Chen et al. (2016), who observed a strong relationship between accuracy and the number of problems in which encoding occurred. Although several explanations exist for this lack of replication, including a low Cronbach’s alpha of .36, the most likely explanation is the difference in the problems used in Chen et al.’s study and our own. The problems in Chen et al.’s study did not increment in difficulty, whereas those in the present study did. Consequently, it is likely that simply encoding a row or a column is insufficient to successfully address the problems in our study, especially the more challenging ones.

On the other hand, as Table 1 shows, accuracy was positively related to integration, *r* =.33, a finding that suggests better problem solvers used integration on more problems than poor problem solvers. This finding supports Hypothesis 2 and is also consistent with those of Chen et al. (2016). It also indirectly supports research that suggests relational reasoning is essential for solving matrix problems (e.g., Dauvier et al., 2014; Resing et al., 2017). That said, caution should be exercised when considering this dependent measure and its accompanying results because it had a low Cronbach’s alpha.

Finally, accuracy was positively related to the number of rows and columns a child examined, a finding that not only supports Hypotheses 3 but also suggests that better problem solvers examined more rows and columns than did poor problem solvers, *r* =.46. This latter finding also supports Novick’s research (1988), which suggests that better problem solvers use the underlying structural features of a problem more so than poor problem solvers. Finally, although integration and the number of rows and columns are complementary measures, based on the aforementioned findings, it appears that the extent to which a child examines the rows and columns for a problem is more predictive of accuracy than just tallying the number of problems in which integration occurs at least once. This conclusion is also supported by the strong relationships between the number of rows and columns and the latency measures for the matrix AOI and the matrix AOI before the first toggle, *r* = .74 and *r* = .62 respectively and the weaker relationships between encoding/integration and the latency measures for the matrix AOI and the matrix AOI before the first toggle, *average r* = .35.

### 3.4 Trade-offs between Matrix and Response-choice AOIs

**Figure 5.**
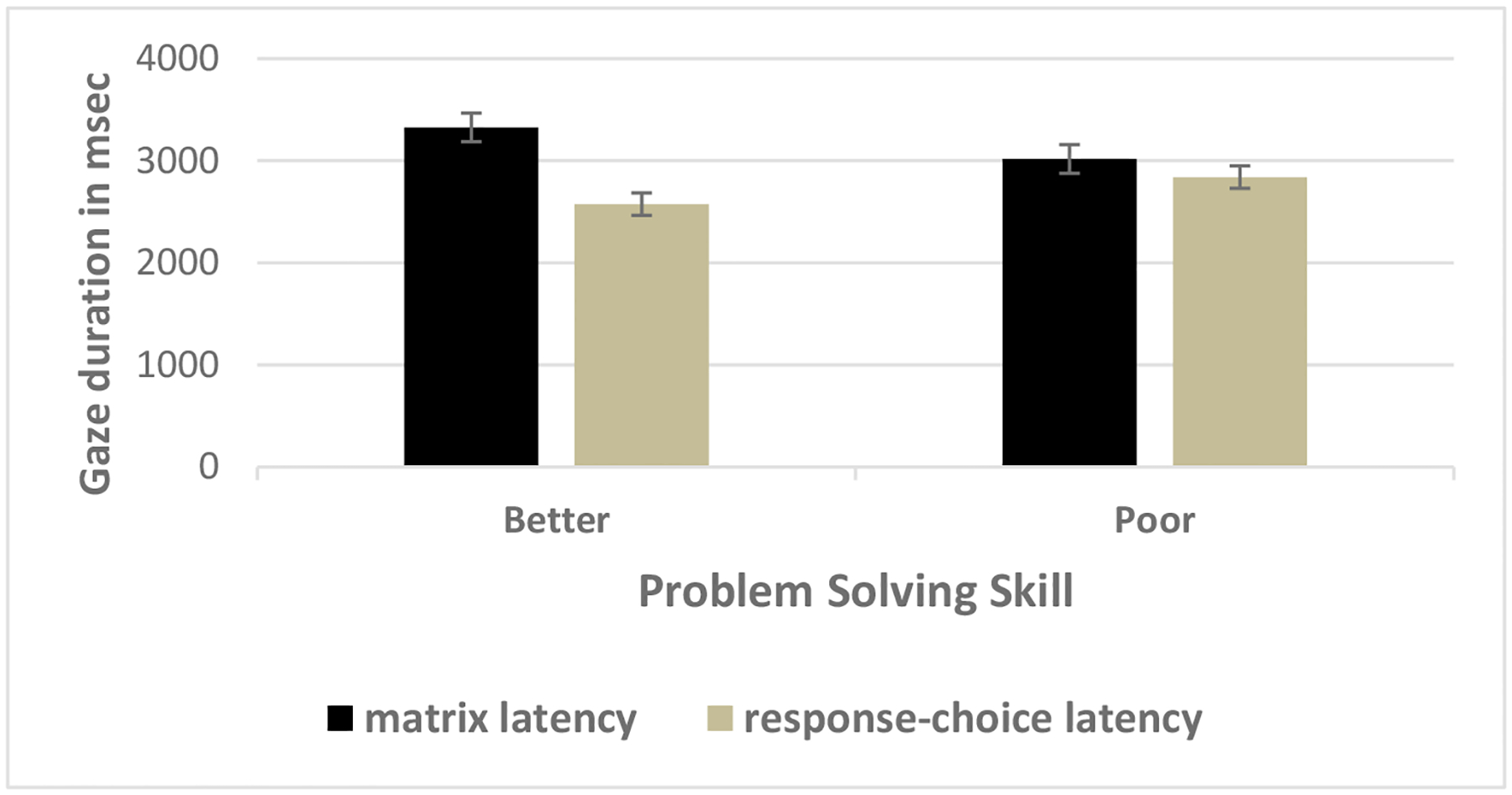
Mean latency for matrix and response choices as a function of problem-solving skill

To gain further insight into children’s relative use of information in the matrix versus the response-choice AOIs, we completed t-tests between mean matrix and response-choice latencies as a function of problem-solving skill. As Figure 5 shows, better problem solvers spent approximately 25% more time on matrix AOIs than response-choice AOIs (3325 versus 2574 *msec*), *t* (36) = 8.00, *p* < .05; a finding that supports the first part of Hypothesis 1 and was replicated using corresponding standard scores, *t* (36) = 3.00, *p* < .05. Moreover, subsequent t-tests revealed that better problem solvers spent more time viewing the matrices before their first toggles than the matrices after their first toggles (1781 msec versus 1544), *t* (36) = 2.12, *p* < .05; a finding that also supports the first part of Hypothesis 1.^Note 2^ Taken as a whole, these findings, in conjunction with the correlational analysis that showed better problem solvers spend proportionally more time on matrices, *r* = .40, but proportionally less time on response choices, *r* =−.40, provide strong evidence that better problem solvers adopt a constructive matching strategy when solving problems.

For poor problem solvers, the pattern of results was different. As Figure 5 shows, although poor problems spent more time on matrix AOIs than response choice AOIs; the difference in their allocation of time between the two AOIs was small, (3018 versus 2839 *msec*), *t* (29) = 2.05, *p* < .05. Moreover, this finding was not supported by corresponding standard scores, which showed that poor problem solvers spent proportionally the same amount of time on matrix and response-choice AOIs, .49 versus .51 respectively, *t* (29) = −1.15, *p* > .05. In addition, poor problem solvers spent less time viewing the matrices before their first toggles than the matrices after their first toggles (1375 msec versus 1642), *t* (29) = −1.83, *p* < .05, a pattern of results that was opposite the better problem solvers.^Note 3^ These findings, in conjunction with the correlational analysis that showed poor problem solvers study matrices for proportionally less time, *r* = .40 but study response choices for proportionally more time, *r* = −.40, suggest that poor problem solvers tend to adopt a response elimination strategy, where they view the matrix for a short time, then spend time on the response choices, only to return to the matrix to view it again.

## 4. Discussion

Vigneau et al. (2006) provided evidence that better adult problem solvers studied matrix AOIs longer than response-choice AOIs, whereas poor adult problem solvers studied response-choice AOIs longer than matrix AOIs. Chen et al. (2016) showed that children who were better problem solvers completed more problems by encoding a row/column and by integrating a row with a column. Novick (1988) showed that better adult problem solvers were more likely to use the structural features of a matrix to solve a problem than poor adult problem solvers. Our novel combination of eye-tracking technology, matrix completion problems, and young problem solvers afforded us a direct and conclusive way to evaluate and extend these findings in multiple ways. First and foremost, when the latencies for matrix and response-choice AOIs were considered, the correlational pattern for the children differed from the pattern observed by Vigneau et al. with adults. Whereas children who were better problem solvers studied matrix AOIs longer than response-choice AOIs, *r* = .35, a finding that is consistent with the adult literature and supports Hypothesis 1, there was no difference in the response-choice latencies for better and poor problem solvers, *r* = −.08, a finding that is inconsistent with the adult literature and does not support Hypothesis 1. However, when the standardized scores were considered, namely, the mean proportion of time on response choices, poor problem solvers studied the response choices longer than better problem solvers. Secondly, better problem solvers studied matrix AOIs longer than poor problem solvers, and the source of this difference was that better problem solvers spent more time studying matrix AOIs before toggling to other areas in the matrix problems. Thirdly, latencies for better and poor problem solvers were equivalent for correct answer AOIs, even when the latencies for correct answers were standardized. Fourthly, better problem solvers studied matrix AOIs longer than response-choice AOIs, whereas poor problem solvers did not. Fifthly, the present results only partially supported Hypothesis 3 and Chen et al.’s results for encoding and integration. In particular, there were no skill differences in the encoding of rows and columns, a finding that is inconsistent with Chen et al. On the other hand, better problem solvers completed more problems using integration (i.e., viewing at least one row and one column) than poor problem solvers, *r* =.33, a finding that is consistent with Chen et al. Finally, better problem solvers studied more rows and columns in the matrix AOIs than the poor problem solvers, a finding that supports Hypothesis 2 by suggesting that children who are better problem solvers use structural features more frequently than poor problem solvers. This finding complements the research of Novick (1998), who showed that adults with better problem-solving skills were more likely to use structural features to solve problems.

### 4.1 Implications for Research and Theory

Two of the more critical findings in the present study concern the proportional time spent on matrices and response choices. Children who were better problem solvers spent more time studying the matrices than did those who were poor problem solvers. These findings reveal qualitative differences between individuals who are poor problem solvers and those who are good problem solvers.

In addition, from a theoretical perspective, our findings provide direct physiological evidence for children’s use of the constructive matching and response elimination strategies. As mentioned in the introduction, constructive matching involves forming an idealized answer using the problems in a matrix before viewing the response alternatives (e.g., Bethell-Fox et al., 1984; Gonthier et al., 2024), and (i) longer latencies on the matrix AOIs before the first toggle and (ii) longer latencies on the matrix AOIs than on the response-choice AOIs are both considered as evidence that the problem solver adopted a constructive matching strategy (Vigneau et al., 2024). They are also hallmarks of better problem-solving skills (Sternberg, 1977). Our findings that: (i) better problem solvers spent more time on the matrix AOIs (i.e., mean matrix latency, mean matrix latency before first toggle, mean proportion time on matrix, mean proportion time on matrix before first toggle) than response AOIs (ii) better problem solvers examined more rows and columns in the matrices than poor problem solvers, (iii) better problem solvers integrated rows and columns on more problems than poor problem solvers, and (iv) better problem solvers spent less time on response choices than poor problem solvers are all evidence that better problem solvers adopted a constructive matching strategy.

On the other hand, response elimination involves spending more time comparing response alternatives in the response-choice AOI to cells in the matrix AOIs (Bethell-Fox et al., 1984; Gonthier et al., 2024), and (i) toggling more frequently between matrix and response-choice AOIs, (ii) shorter latencies on the matrix AOIs, and (iii) shorter latencies on matrix AOIs before toggling to response-choice AOIs are considered as evidence that the problem solver adopted a response elimination strategy (Gonthier et al., 2024). The present study did not find a relationship between problem-solving skill (i.e., accuracy) and toggling between matrix and response-choice AOIs; however, we did observe that poorer problem solvers spent less time than better problem solvers on matrix AOIs (i.e., mean matrix latency, mean matrix latency before first toggle, mean proportion time on matrix, mean proportion time on matrix before first toggle), and more time than better problem solvers on response-choice AOIs. Moreover, although poor problem solvers tended to divide their time equally between the matrices and response-choice AOIs, the bulk of their time on the matrices was after they had looked at response choices. So, although poor problem solvers did not toggle disproportionately between response choices and the matrices, they returned to the matrices for a prolonged amount of time to re-study them after viewing response choices. Combined, these findings suggest that individuals with poor problem-solving skills tend to adopt a response-elimination strategy. They move quickly to the response choices, but then they seem to understand that there is a need to acquire more information and therefore return to the matrix. However, their lack of scanning of rows and columns in the matrices suggests they are not adopting a constructive matching strategy.

Finally, our finding that better problem solvers scan rows and columns more frequently than poor problem solvers complements recent research examining transitions in problem solving. According to this research, complex problem-solving involves three stages: (i) an examination stage, where a problem-solver explores the problem in order to generate knowledge about a problem, (ii) a problem representation stage, where the problem solver builds a mental representation of the problem, and (iii) a knowledge use stage, where the problem solver uses the knowledge and applies it to other problems (Molnar & Greiff, 2023). Research also suggests that understanding a problem and depicting its structure are crucial in problem solving (Molnar & Greiff, 2023). The present findings concerning the use of rows and columns assess the first stage of this model. They also support the idea that understanding a problem and depicting its structure are crucial for problem-solving.

### 4.2 Limitations

We labeled children with strong problem-solving skills as better problem solvers rather than skilled problem solvers. We made this choice because the word *skilled* implies years of experience, which 7–8-year-old children don’t have. Nor do most children have experience with matrix completion problems. Nevertheless, we did not inquire about our children’s histories with puzzles or games, which require using structural features, and therefore it is possible that some of the children had some experience with the abstraction of underlying features, albeit minimal. This is something that future research may wish to explore. We are inclined to suggest that some aspects of problem-solving, such as using structural features, may be innate.

Additionally, although we have mentioned that some of our findings are related to research examining relational reasoning, we did not directly assess or examine relational reasoning, as such goals are beyond the scope of this study. Nevertheless, future research may wish to pursue this avenue, although we strongly recommend the inclusion of a measure of relational reasoning that is external to the matrix reasoning task.

Other study limitations are the age of our participants and our measure. The present study was limited to a sample of 7- to 8-year-olds and one measure of matrix problem solving. Future research should seek to generalize the present findings by using participants of other ages and other measures. In addition, the present study did not assess working memory or cognitive speed, two other constructs known to be predictive of matrix tasks, such as the one used in the present study. Consequently, whether the present findings are independent or interdependent with working memory and cognitive speed is unclear.

In summary, given the pivotal role that eye movement paradigms play in reading (Reingold et al., 2001), it is surprising that few studies have used this technology with problem-solving. The present study illustrates that eye movement paradigms help supplement traditional paper and pencil measures of problem-solving, even when the participants are children (Reingold et al., 2001). Moreover, from a practical perspective, the present study demonstrates that many children possess problem-solving skills that are as sophisticated as those of many adults.

## Figures and Tables

**Figure 4(i):**
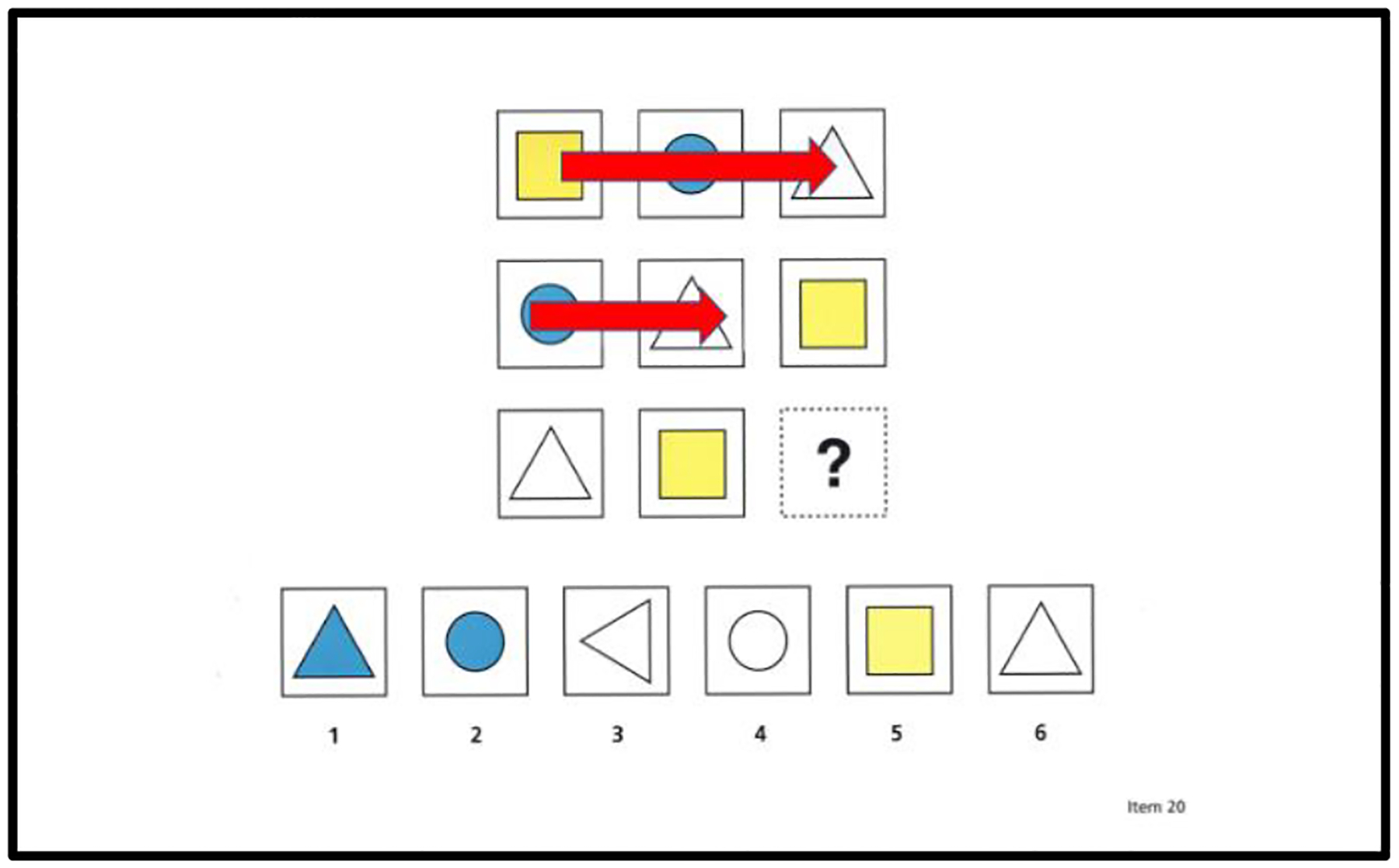
Examples of full and partial scans of rows

**Figure 4(ii).**
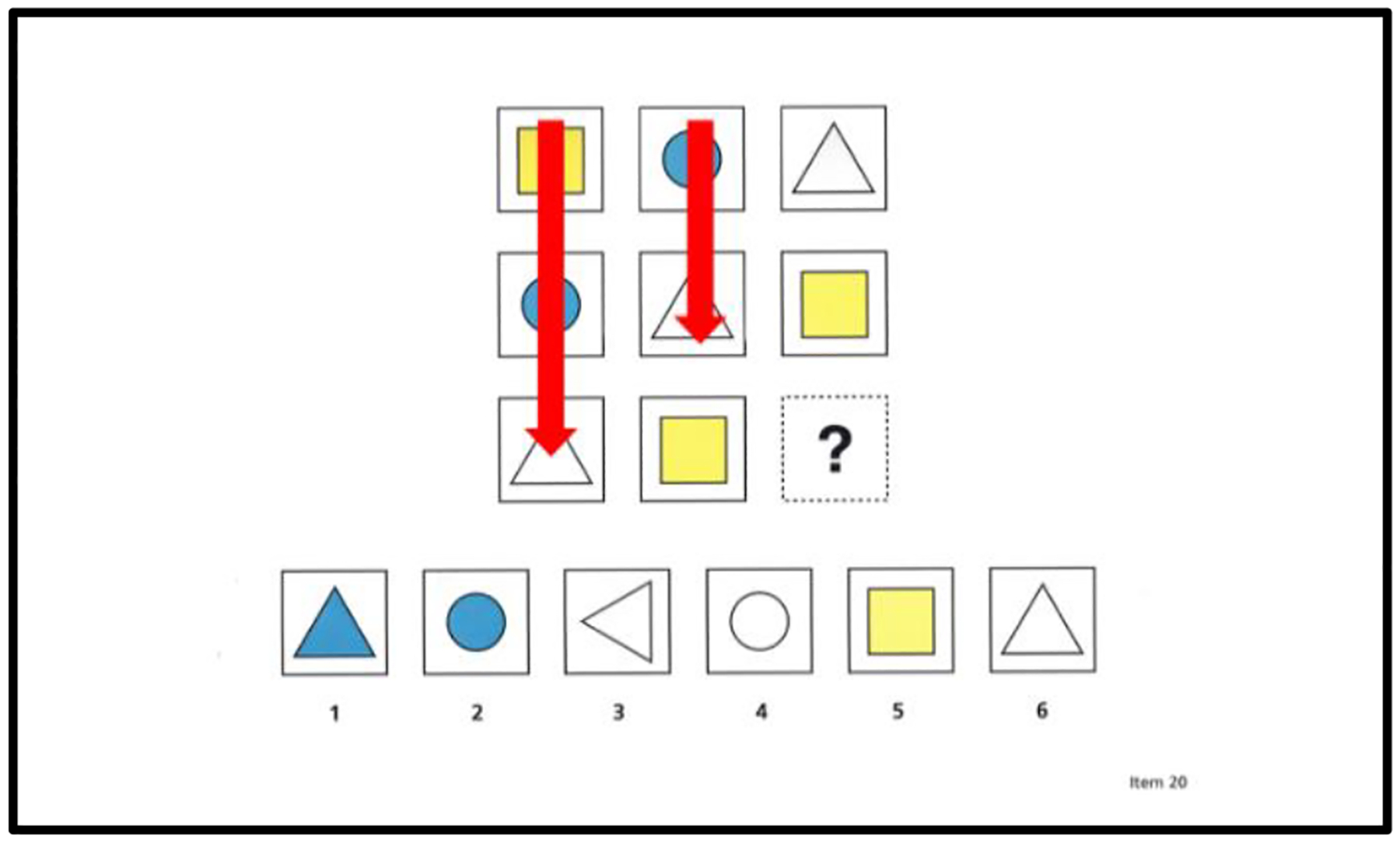
Examples of full and partial scans of columns

**Table 1.** Correlations among Number of Problems Answered Correctly and Eye-tracking Measures (*N* = 67)

	1	2	3	4	5	6	7	8	9	10	11	12	13	14	15	16	17	18	19
1. Accuracy - 22 problems	---	.18	.35*	.45*	.05	.40*	.33*	−.11	−.08	.16	−.21	−.40*	.09	−.50*	.06	−.07	.18	.33*	.46*
2. Mean latency		---	.94*	.58*	.75*	.21	−.09	.27*	.89*	.81*	.82*	−.21	−.17	−.10	.57*	−.17	.24*	.40*	.65*
3. Mean matrix latency			---	.71*	.71*	.48*	.12	.21	.68*	.65*	.60*	−.48*	−.26*	−.32*	.48*	−.21	.28*	.47*	.74*
4. Mean matrix latency before first toggle				---	.02	.56*	.67*	−.42*	.31*	.38*	.22	−.56*	−.25*	−.40*	−.05	−.53*	.26*	.38*	.62*
5. Mean matrix latency after first toggle					---	.13	−.50*	.72*	.66*	.55*	.64*	−.13	−.12	−.05	.73*	.23	.14	.29*	.44*
6. Mean prop time on matrix						---	.60*	−.02	−.17	−.13	−.17	−1/0	−.46*	−.73*	.08	−.07	.14	.40*	.36*
7. Mean prop time on matrix before first toggle							---	−.81*	−.34*	−.26*	−.35*	−.60*	−.29*	−.43*	−.39*	−.38*	.14	.10	.17
8. Mean prop time on matrix after first toggle								---	.30*	.24	.31*	.02	.03	−.01	.54*	.42*	−.08	.18	.06
9. Mean response choice latency									---	.86*	.95*	.17	−.03	.20	.58*	−.08	.15	.24*	.41*
10. Mean correct answer latency										---	.66*	.13	.32*	−.11	.41*	−.20	.18	.34*	.45*
11. Mean incorrect response choice latency											---	.17	−.23	.36*	.60*	.01	.10	.15	.32*
12. Mean prop time on response choices												---	.46*	.73*	−.08	.07	−.14	−.40*	−.36*
13. Mean prop time on correct answer													---	−.27*	−.17	−.09	−.01	−.06	−.05
14. Mean prop time on incorrect response choices														---	.04	.14	−.14	−.39*	−.36*
15. Mean number of toggles															---	.70*	−.02	.17	.21
16. Mean rate of toggling																---	−.25*	−.14	−.31*
17. Encoding																	---	.46*	.62*
18. Integration																		---	.59*
19. Mean number of rows and columns																			---
Mean	18.71	5875.47	3087.38	1599.29	1588.10	0.51	0.30	0.21	2688.09	1135.15	1552.94	0.49	0.25	0.25	3.56	0.61	8.82	3.02	3.25
Standard deviation	1.97	1341.35	816.07	571.93	571.95	0.04	0.07	0.06	645.54	270.37	436.28	0.04	0.03	0.04	1.05	0.15	2.79	1.59	1.22
Minimum	14.00	3715.36	1760.87	528.18	579.84	0.40	0.13	0.07	1645.88	696.30	869.83	0.36	0.17	0.15	1.68	0.31	2.00	0.00	0.77
Maximum	22.00	10657.62	5632.92	3254.05	2939.37	0.64	0.48	0.33	5025.71	2021.46	3182.31	0.60	0.32	0.35	6.79	1.01	14.00	7.00	6.49
Skew	−0.33	1.02	0.71	0.63	0.23	0.05	−0.28	−0.10	1.09	1.12	0.90	−0.05	−0.11	0.40	0.56	0.51	0.38	−0.40	0.91
Kurtosis	−0.49	1.87	0.57	0.57	−0.24	0.80	−0.11	−0.35	1.86	1.54	1.66	0.80	0.38	0.23	0.17	−0.00	−0.10	−0.30	−0.07
Cronbach alpha	.65	.73	.71	.79	.47	.68	.64	.44	.69	.78	.70	.68	.68	.47	.71	.53	.36	.60	.70

## Data Availability

The data that support the findings of this study are available on request from the corresponding author. The data are not publicly available due to privacy or ethical restrictions.

## Footnotes

Note 1. Five problems were removed because they did not have any obvious structured row or column pattern.

Note 2. Although the Cronbach alpha is low for the mean matrix after the first toggle, the interpretation of the results reported in this section is not solely based on this measure.

Note 3. Although the Cronbach alpha is low for the mean matrix after the first toggle, the interpretation of the results reported in this section is not solely based on this measure.

## Data sharing statement

No additional data are available.
